# Changes in Antimicrobial Use Prevalence in China: Results from Five Point Prevalence Studies

**DOI:** 10.1371/journal.pone.0082785

**Published:** 2013-12-23

**Authors:** Chunhui Li, Nan Ren, Ximao Wen, Pengcheng Zhou, Xun Huang, Ruie Gong, Yixin Lv, Li Feng, Hongman Wu, Zhenru Liu, Chenchao Fu, Xin Huang, Jie Li, Yuhua Chen, Cui Zeng, Shuangyan Zuo, Xinrui Xiong, Xiuhua Xu, Anhua Wu

**Affiliations:** Infection Control Center, Xiangya Hospital, Central South University, Changsha, China; University of Maryland, School of Medicine, United States of America

## Abstract

**Objective:**

The abuse of antimicrobials is a serious concern in China. Several measures have been taken to improve the rational use of antimicrobials, including the establishment of a national surveillance network for antimicrobial use. This study describes the dynamic changes in antimicrobial use in China between 2001 and 2010, with the scope of identifying targets to improve the prescription of antimicrobials.

**Methods:**

Five point prevalence surveys were performed in hospitals across mainland China in 2001, 2003, 2005, 2008, and 2010. All inpatients who were admitted for at least 24 hours were included in the study. Details regarding antimicrobial use by these patients and the collection of samples for bacterial culture from inpatients administered therapeutic antimicrobials were recorded.

**Results:**

The surveys encompassed tertiary hospitals from all 31 provinces of mainland China. Antimicrobial use prevalence decreased from 54.79% in 2001 to 46.63% in 2010. While this decline was observed in most hospital departments, antimicrobial use remained stable or increased in others. Antimicrobial use prevalence was relatively high in the Pediatrics departments and general intensive care units, whereas it was lower in the Obstetrics (Neonatal group) departments in each survey. The proportion of patients administered a single antimicrobial increased from 60.78% in 2001 to 70.16% in 2010, while the proportion of administration of two or more antimicrobials declined. The bacterial culture rate increased from 25.22% in 2003 to 34.71% in 2010. Antimicrobial use prevalence (47.96% vs 46.16%), bacterial culture rate (36.40% vs 34.19%), and the proportion of administration of a single antimicrobial (71.41% vs 67.33%) were higher in teaching hospitals than in nonteaching hospitals in 2010.

**Conclusion:**

Although measures for enhancing the rational use of antimicrobials have been effective, further improvements are required. The findings from this study can promote such improvements.

## Introduction

Antimicrobial abuse is increasingly becoming an issue of concern worldwide. Such misuse not only results in excessive treatment costs for each patient but also causes numerous public health issues [1], [2], [3]. The most important one is the high prevalence of antimicrobial resistance attributed to the frequent use of antimicrobials [4], [5], [6], [7], [8], which presents a serious threat to patients and increases morbidity and mortality [9], [10].

Therefore, monitoring antimicrobial use has become increasingly important. However, data describing antimicrobial use in hospitals has only become available since the turn of the century. Since this point in time, many systems for the surveillance of antimicrobial use have been established [4], including the European Surveillance of Antimicrobial Consumption (ESAC) [4], [11], the Swedish Strategic Programme Against Antibiotic Resistance [12], and the Danish Integrated Antimicrobial Resistance Monitoring and Research Programme [13]. Of these, ESAC, which was established in 2000 and contained 172 participating hospitals in 2009, is thought to be one of the most successful surveillance systems for antimicrobial use in the world. It aimed to identify targets for quality improvement in antimicrobial prescription on the basis of web-based point prevalence surveys (PPSs) and established a set of standardized methods for antimicrobial use surveillance [4], [14]. ESAC has generated several annual reports and proposed pertinent suggestions, thus improving the rational use of antimicrobials in Europe.

China is considered the world's largest antimicrobial consumer, with the most serious antimicrobial resistance problems in the world [15]. This is because surveillance and regulation of antimicrobial use were essentially absent in mainland China before 2000. In 2000, however, the Chinese Ministry of Health (MOH) committed to decrease the improper use of antimicrobials and strengthen efforts to control their use. A surveillance network of antimicrobial use based on the National Healthcare-associated Infection Surveillance System (NHAISS) was well established in 2001. The infection control center of the Xiangya Hospital at Central South University was authorized by the MOH to supervise the system. Additional measures, including the issue of “principles for the clinical use of antibiotics”, were taken by the MOH in 2004 in order to improve the rational use of antimicrobials [16], [23]. Furthermore, the local government and hospitals created additional rules for antimicrobial use subsequent to the implementation of this document. Nevertheless, there are few publications describing baseline data and the dynamic changes in antimicrobial use since 2000 in mainland China. Here we describe the results of 5 PPSs performed by the NHAISS to characterize the general status of antimicrobial use in China at a series of time points from 2001 to 2010. The analysis of this data will enable an assessment of the dynamic changes in antimicrobial use in China, with the scope of identifying targets for improving antimicrobial prescription and evaluating the effects of the abovedescribed measures against antimicrobials abuse.

## Methods

### Ethics statement

This study was designed and conducted in accordance with the ethical principles of the Chinese Ministry of Health, which authorized the study, and approved by the Ethical Committee of the Xiangya Hospital, Central South University. The ethical certification has been supplied with the supplementary documents. The research was not conducted outside of China. This was an observational study and not a clinical trial; therefore, it did not interfere with the treatment of patients and posed no risk to participants. As approved by the Ethical Committee of the Xiangya Hospital, informed consent was verbally obtained from patients and recorded it on the questionnaires.

### Hospitals

The study was performed in tertiary hospitals across Mainland China. Eligible hospitals were required to have established healthcare-associated infection (HAI) management committees and sufficient staff for hospital infection prevention and control, with at least one professional per 250 beds.

### Inquiring officers

The key inquiring officers in charge of the survey in a hospital were predominantly from the HAI department and were trained in PPS methods. Such officers were qualified by the national or provincial Hospital Infection Training Centre; and then they train additional inquiring officers at their hospitals. Each hospital had several survey teams, and each survey team had 4–5 inquiring officers, including attending physicians, infection professionals, and clinical pharmacists.

### Data collection

Data on antimicrobials use, which were principally collated from medical records including clinical data, laboratory tests, and other examinations, were collected by the dedicated survey groups at a given hospital. When required, inquiring officers could request additional information from doctors and nurses. Verbal informed consent was obtained at the initiation of the surveys. Then, the attending physicians visited patients at the bedside while other staff members reviewed medical records and completed the questionnaire. Patient information was confirmed by two different inquiring officers. Antituberculosis drugs; antiviral agents; antifungal agents; antimicrobials administered by aerosol; antimicrobial eye, ear, or nose drops; and antimicrobials applied directly to the skin or wounds were excluded from the surveys. Antimicrobials were classified into three groups on the basis of the purposes of use: therapeutic, prophylactic, or therapeutic+prophylactic (both). Combination treatment was defined as the use of two or more antimicrobials at the same time on the day of the survey. All inpatients who were admitted for at least 24 hours and presented at 8 am on the day of the survey were included. Patients due to receive antimicrobials on the day of the survey were identified and the details of prophylaxis and/or therapy were recorded on their data sheet. Information pertaining to the availability of specimens bacterial culture was documented for any patient prescribed therapeutic or therapeutic + prophylactic antimicrobials.

Preliminary data from the first three surveys (2001, 2003, 2005) were collected using paper questionnaires and posted to the NHAISS office. Since 2008, data was submitted by all hospitals using a web-based system, namely the National Healthcare-Associated Infection Control Office Automation System (http://oa.yygr.cn/index.asp). All initial and repeated test results were recorded and are freely accessible to researchers at the surveyed hospitals by their accession number.

### Statistical analysis

The antimicrobial use prevalence (AUP) was determined by the following equation: number of patients prescribed antimicrobials/number of patients surveyed ×100%. The Bacterial Culture Rate (BCR) was determined as follows: number of patients whose samples were sent to the laboratory for bacterial culture/number of patients administered antimicrobials for therapy and therapy + prophylaxis. Differences in proportions and ratios between sets of data were determined by the chi-square test using STATA 8.2 (Stata Corporation, College Station, Texas, USA). A *P*-value of <0.05 was considered statistically significant. Standard methods were used to calculate 95% confidence intervals for proportions and ratios.

## Results

### Hospitals and Patients

The total number of tertiary hospitals in mainland China was 977 in 2001, 962 in 2003, 946 in 2005, 1196 in 2008, and 1284 in 2010. The number of surveyed hospitals was 82 in 2001, and it increased to 247 in 2010.The number of hospitals surveyed in 2003, 2005, and 2008 were 77, 86, and 139, respectively. The total number of inpatients that met the inclusion criteria were 74670 in 2001, 65656 in 2003, 81402 in 2005, 136402 in 2008, and 269328 in 2010. More than 97% inpatients that met the inclusion criteria accepted participate in each survey. The number of surveyed patients were 73228 in 2001, 64126 in 2003, 79986 in 2005, 134192 in 2008, and 265342 in 2010. The surveyed hospitals were selected from all 31 provinces of mainland China and included teaching and nonteaching hospitals, comprehensive hospitals and specialty hospitals (i.e. infectious disease hospitals, cancer hospitals, pediatric hospitals, and maternity and childcare hospitals).

### AUP

**Figure 1:**
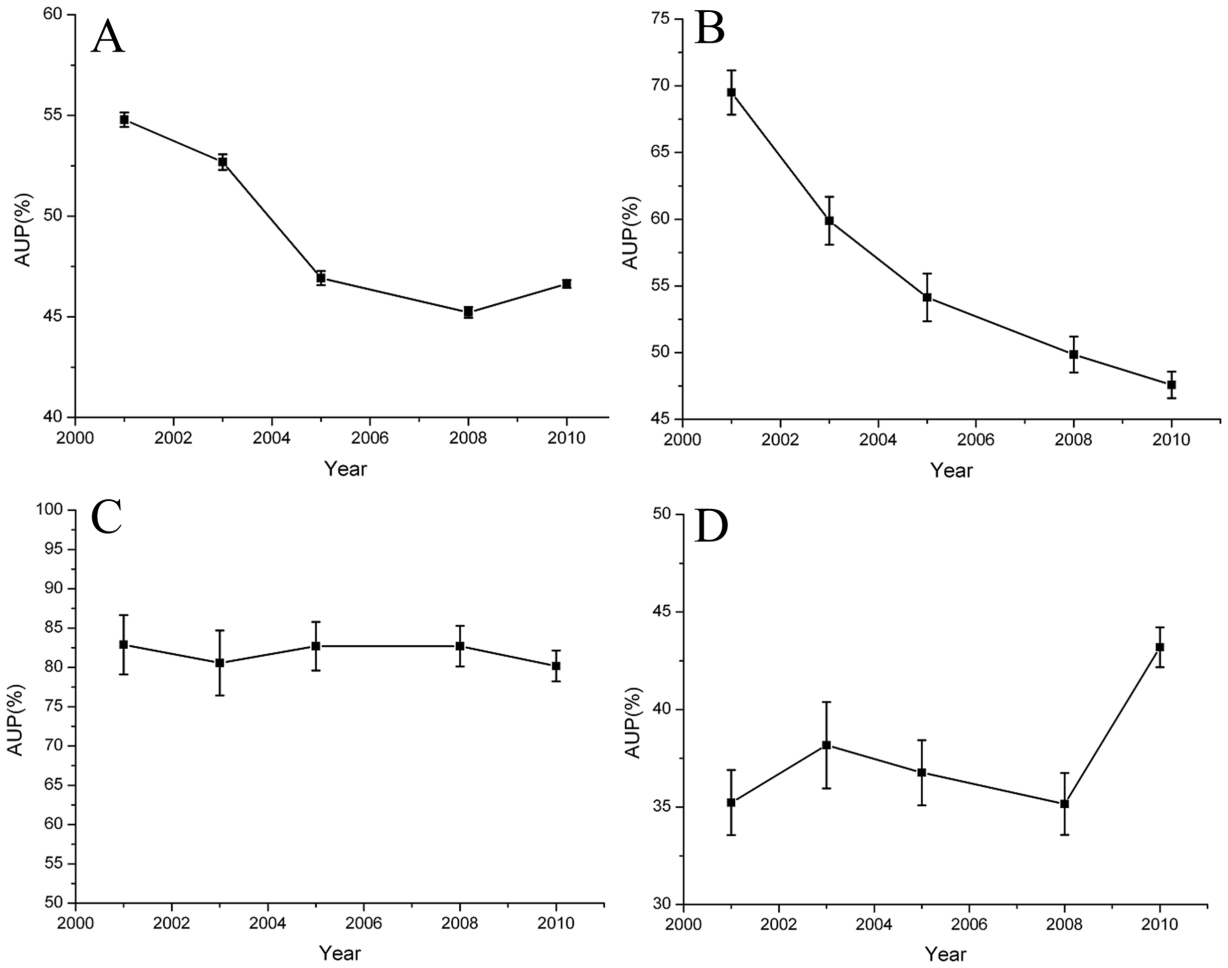
Changes in antimicrobial use prevalence (AUP; 95% confidence intervals) from 2001–2010. A. The overall AUP of Chinese hospitals decreased over time. B. The AUP of the Neurosurgery departments decreased over time. C. The AUP of the general intensive care units remained stable. D. The AUP of the Infectious Diseases departments increased over time.

The overall AUP from the five surveys was 47.96%, and it decreased over time after 2001. Specifically, the AUP of surveyed hospitals ranged from 35.21% to 78.58%, with the average being 54.79% in 2001. The average AUP decreased to 52.68%, 46.92%, and 45.21% in 2003, 2005, and 2008, respectively, and it slightly increased (46.63%) in 2010 (Figure 1A). The AUP showed different distributions for different hospital departments at baseline and exhibited a dynamic trend. While a decline was observed in most departments (e.g., neurosurgery; Figure 1B), it remained constant and high in the general intensive care units (ICUs; Figure 1C) and Pediatrics (non-neonates) departments, even showing an increase in the Infectious Diseases departments (Figure 1D). The AUP for the Pediatrics departments (both neonatal and non-neonatal groups), general ICUs, and Respiratory departments were ranked in the top five in each survey (Table 1). Furthermore, the AUP for the Surgical Urology; Burns; and Ear, Nose, and Throat departments were also relatively higher than those for other departments in each survey (Table 1). In contrast, the AUP for the Obstetrics (Neonatal group), Neurology, Endocrinology, and Traditional Chinese Medicine departments were relatively lower in each survey (Table 1).

**Table 1:** Antimicrobial use prevalence in each department in each survey.

Departments	Number of surveyed patients (antimicrobial use prevalence; %; 95% confidence intervals)				
	2001	2003	2005	2008	2010
**Internal medicine**					
Respiratory medicine	3589(79.38,78.03–80.67)	2836 (83.00, 81.57–84.34)	4123 (75.55, 74.21–76.84)	6863 (76.32, 75.30–77.31)	12152 (73.23, 72.44–74.01)
Endocrinology	1689(30.31, 28.16–32.54)	1269 (34.52, 31.95–37.18)	2268 (25.49, 23.74–27.32)	3356 (22.14, 20.77–23.58)	8954 (21.58, 20.74–22.44)
Infectious diseases	3156 (35.23, 33.58–36.91)	1852 (38.17, 35.98–40.40)	3196 (36.76, 35.11–38.45 )	3498 (35.16, 33.59–36.76)	8952 (43.19, 42.17–44.22)
Neurology	3566 (30.26, 28.77–31.79)	3947 (36.48, 34.99–37.99)	5191 (20.48, 19.40–21.60)	9589 (20.01, 19.22–20.82)	14574 (18.98, 18.35–19.62)
Traditional Chinese medicine	1981 (35.08, 33.01–37.21)	1569 (35.18, 32.86–37.58)	1852 (28.62, 26.61–30.72)	1978 (28.16, 26.22–30.18)	5741 (25.45, 24.34–26.59)
**Surgery**					
Surgical urology	2521 (72.23, 70.45–73.94)	1368 (72.15, 69.72–74.46)	2989 (66.88, 65.17–68.54)	4935 (65.53, 64.19–66.84)	9841 (68.47, 67.54–69.38)
Burns	687 (73.65, 70.23–76.81)	1589 (69.86, 67.56–72.07)	895 (60.89, 57.65–64.03)	789 (59.19, 55.72–62.57)	1854 (58.20, 55.94–60.43)
**Obstetrics**					
Adult group	1980 (66.52, 64.41–68.56)	1657 (67.83, 65.54–70.04)	1774 (56.99, 54.67–59.28)	3952 (58.12, 56.57–59.65)	13487 (58.17, 57.34–59.00)
Neonatal group	771 (28.15, 25.09–31.43)	852 (21.60, 18.97–24.49)	521 (17.47, 14.45–20.97)	451 (15.52, 12.47–19.15)	1245 (8.59, 7.16–10.28)
**Pediatrics**					
Neonatal group	501 (82.44, 78.87–85.52)	689 (83.89, 80.96–86.45)	687 (72.63, 69.18–75.83)	1689 (78.51, 76.49–80.40)	3607 (75.52, 74.09–76.90)
Non-neonatal group	2592 (81.79, 80.26–83.23)	2123 (81.58, 79.87–83.17)	3258 (76.58, 75.10–78.00)	5129 (80.02, 78.90–81.09)	9874 (82.17, 81.40–82.91)
**ENT**	2177 (70.60, 68.65–72.48)	1968 (72.56, 70.55–74.49)	1458 (70.23, 67.83–72.52)	2241 (69.34, 67.40–71.21)	6508 (67.12, 65.97–68.25)
**General ICU**	380 (82.89, 78.78–86.34)	350 (80.57, 76.10–84.37)	578 (82.70, 79.40–85.57)	821 (82.7, 0,79.96–85.13)	1589 (80.18, 78.15–82.07)
**Other departments**	2672 (38.14, 36.32–40.00)	1689 (28.36, 26.26–30.56)	2896 (27.87, 26.27–29.53)	3723 (21.46, 20.17–22.81)	9874 (12.78, 12.14–13.45)

ENT, Ear, Nose and Throat Department. The table presents the data of key departments due to space restraints. For full details, refer to Table S1.

### Purposes of Antimicrobial and Combination Therapy Use

**Figure 2:**
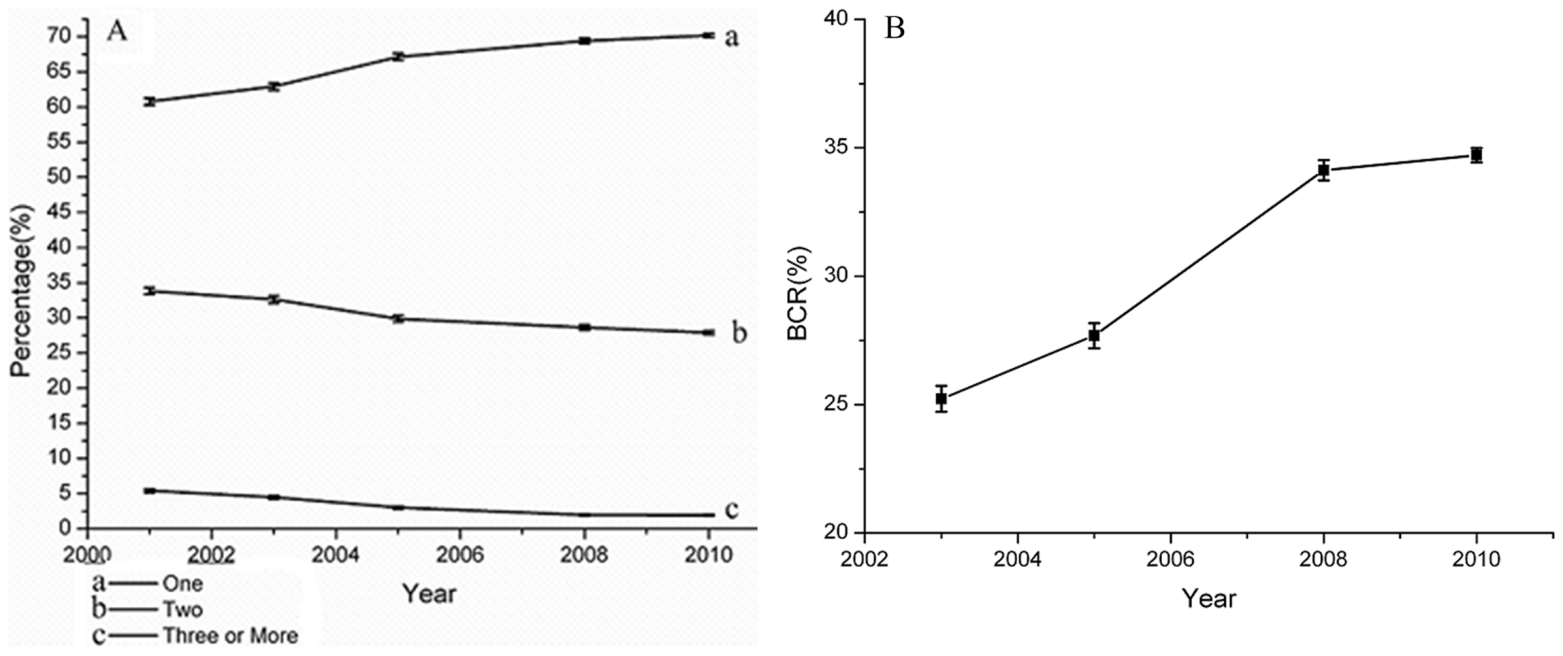
Changes in the proportion of single or combination antimicrobial therapy (with 95% confidence intervals) (A) and the bacterial culture rate (BCR, with 95% confidence intervals) (B). A. Administration of a single antimicrobial increased while that of two or more antimicrobials declined over time. B. The overall BCR of Chinese hospitals increased over time.

The purposes of antimicrobial therapy use did not change much between 2003 and 2010, although statistically significant differences were observed. Specifically, in each survey, 47.82%–49.23% and 36.53%–42.13% patients were administered antimicrobials for therapeutic and prophylactic purposes, respectively. The proportion of patients administered antimicrobials for therapy + prophylaxis decreased from 15.65% in 2003 to 8.64% in 2008, with a small increase in 2010 (10.69%). In terms of antimicrobial combination therapy, the proportion of patients administered a single antimicrobial increased significantly from 60.78% in 2001 to 70.16% in 2010 (Figure 2A). At the same time, the proportion patients administered two and three or more antimicrobial declined from 33.82% to 27.91% and from 5.40% to 1.93%, respectively (Figure 2A).

### BCR

Among patients administered antimicrobials for therapy as well as therapy and prophylaxis in 2003, 25.22% had their samples sent to the laboratory for bacterial culture. This rate increased to 27.68%, 34.12%, and 34.71% in 2005, 2008, and 2010, respectively (Figure 2B).

### 2010 Survey Details: Teaching vs Nonteaching Hospitals

In total, of the 247 hospitals surveyed (total 265342 surveyed inpatients; mean, 1106; median, 970; range, 501–4708) in 2010, 41 were teaching hospitals while the rest were nonteaching hospitals. The AUP for the teaching hospitals (47.96%) was higher (*P*<0.05) than that for the nonteaching hospitals (46.16%; Table 2). The same was observed for the BCR, with teaching hospitals having a higher rate (36.40%) than nonteaching hospitals (34.19%; *P*<0.05; Table 2). In contrast, the proportion of antimicrobial use for therapy was higher in nonteaching hospitals (51.89%) than in teaching hospitals (41.17%; *P*<0.05; Table 2), while antimicrobials used for prophylaxis were more widely applied in teaching hospitals than in nonteaching hospitals (48.02% vs 37.47%; *P*<0.05; Table 2). With regard to combination antimicrobial therapy, the proportion of patients administered a single antimicrobial was higher in teaching hospitals (71.41%) than in nonteaching hospitals (67.33%; *P*<0.05; Table 2), while the proportion of patients administered two antimicrobials was lower in teaching hospitals (26.25%) than in nonteaching hospitals (30.81%; *P*<0.05; Table 2).

**Table 2:** Antimicrobial use at teaching and nonteaching hospitals in 2010.

Type of hospital	Number of surveyed hospitals	Number of surveyed patients	Antimicrobial use prevalence (%; 95% confidence intervals)	Proportion of antimicrobial use purpose (%)			Proportion of antimicrobial combination therapy use (%)			BCR (%)
				Therapy	Prophylaxis	Both	One	Two	Three or more	
Teaching hospital	41	69815	47.96 (47.59–48.33)	41.17	48.02	10.81	71.41	26.25	2.34	36.40
Nonteaching hospital	206	195527	46.16 (45.94–46.38)	51.89	37.47	10.64	67.33	30.81	1.86	34.19

## Discussion

Improving the rational use of antimicrobials is a priority for Chinese health administrative departments, and effective and reasonable surveillance of antimicrobial use is the cornerstone of this project [1]. With the strong support of the Chinese Ministry of Health, NHAISS staff have committed to improve PPS methods and data submission techniques over the last 12 years. Five national PPSs of antimicrobial use have been performed and representative and reliable data detailing the antimicrobial use of hospitalized patients has been obtained. In general, hospitals and districts involved in the surveys increased over the duration of the surveys. Notably, 247 hospitals participated in the survey in 2010, which encompassed almost all the provinces in mainland China, whereas the number of participating hospitals in the ESAC PPS was 172 in 2009 [4]. This result demonstrates that a feasible surveillance system for antimicrobial use in a large sample of hospitals in mainland China has been successfully put into practice.

As previously mentioned, the irrational use of antimicrobials is a serious problem in China, as typically characterized by a high AUP, antimicrobials administered in combination without any specific indications, and antimicrobials administered without concomitant pathogen culture. This study showed that measures introduced by Chinese health administrative departments and hospitals have successfully contained irrational antimicrobial use. This was demonstrated by the decrease in the AUP and the proportion of combination antimicrobial therapy and the considerable increase in the BCR. In particular, these changes were most notable in 2005, following the publication of the “principles for the clinical use of antibiotics” in 2004.

However, when compared with that in other countries, the AUP for China was similar to that for other developing countries and relatively higher than that for developed countries. According to the ESAC report, the AUP for European countries was 30.1% in 2006 and 29.0% in 2009 [4], [11]. Another survey revealed that the AUP for adult acute-care hospitals in Canada was 36.3% in 2002 [17]. In contrast, it was 55.4% in Brazil in 2004 and 44.0% in Malaysia in 2001 [18], [19]. The disease spectrum in China is different from that in developed countries and the incidence of infectious diseases is still high [20]; further more Lagging administrational regulation of antimicrobial use in China, in combination with the strict administration of and guidelines for antimicrobial use in developed countries, may explain the current position of China with respect to antimicrobial misuse. Pathogen culture can introduce significant improvements by avoiding the use of broad-spectrum antimicrobials for extended periods and, consequently, decreasing the emergence of drug resistance [21]. Compared with the BCR in Europe (43% for adult patients and 54% for children in 2006) [11], the BCR was much lower in China (34.71% in 2010). Taking this into account, the AUP and BCR for Chinese hospitals can be further improved.

The AUP showed a different distribution among different departments at baseline and exhibited a dynamic trend, providing useful data for the preparation of new policies on antimicrobial use and identification of new surveillance targets. Because the incidence of infection was higher in the Pediatrics departments (both non-neonatal and neonatal), general ICUs, and Respiratory Medicine departments, the AUP for these departments were relatively higher than that for other departments. Therefore, a higher AUP target should be permitted in these departments. In line with this consideration, the targeted rate of antimicrobial use should be lower in the Obstetrics (neonatal group), Neurology, Endocrinology, and Traditional Chinese Medicine departments. Although the rate of antimicrobial use decreased in most departments, it remained stable in the Pediatrics departments (neonatal) and General ICUs, even increasing in the Infectious Diseases departments. Of particular note, most patients were admitted to the Infectious Diseases departments because of viral hepatitis, not bacterial infection. Antimicrobial use in these departments requires further assessment in order to identify the reasons for their high rate of antimicrobial use, particularly the rate of irrational antimicrobial use, which was not assessed in the surveys in the present study. These departments should be focal points for future surveillance.

The present study found that the AUP and the proportion of antimicrobial use for prophylaxis was higher in teaching hospitals than in nonteaching hospitals. Generally, teaching hospitals provide better medical services and admit patients who are more severely ill compared with nonteaching hospitals in China; therefore, it is reasonable to presume that the incidence of infection and the proportion of patients with immunodeficiency or those requiring complex surgery may be higher in the former than in the later. The present study also demonstrates that teaching hospitals have a better performance in terms of the rational use of antimicrobials compared with nonteaching hospitals. This is exemplified by the higher BCR and lower proportion of combination antimicrobial therapy. Therefore, nonteaching hospitals should also be a focus for future incentives designed to further improve the rational use of antimicrobials in China.

It is worth mentioning that, compared with the data from 2008, several indices related to the rational use of antimicrobials, including the overall AUP and AUP for some departments, deteriorated in 2010, 6 years after the last large-scale antimicrobial use improvement campaigns in 2004. Fortunately, realizing the serious consequences of antimicrobial misuse, Chinese health administrative departments have been actively working to improve the current situation. “Administrative regulations for the clinical use of antimicrobial agents”, considered the most rigid regulations controlling the prescription of antimicrobials till date, was implemented on August 1, 2012 [22], [23]. According to these regulations, all antimicrobials must be categorized into three classes: nonrestricted, restricted, or special-grade. The authority to prescribe a given antimicrobial is dependent on the position and professional title of a physician. In addition, the health-care authority regularly publishes information on the use of antimicrobial agents in hospitals, commends physicians who closely follow the regulations, and imposes penalties on medical staff who violate these regulations. Furthermore, the number of different antimicrobials available for use in a given institution is limited. In this light, no more than 50 antimicrobials are allowed to be used in a tertiary hospital, and up to 35 different antimicrobials are permitted to be used in a secondary or specialty hospital. Moreover, many hospitals assign a target rate of antimicrobial use for each department in order to achieve their overall target rate. The surveys have made it possible to set important targets for improving and monitoring antimicrobial use and generating baseline data for future comparisons in the antimicrobial use improvement campaign. It is expected that the implementation of the new regulations will further decrease antimicrobial misuse in China.

## Conclusions

Measures for enhancing the rational use of antimicrobials in clinical practice were suggested to be effective in the first decade of the 21st century in China. In addition to the increase in the BCR, the overall AUP, the AUP of most hospital departments and the proportion of combination antimicrobial therapy decreased considerably. However, the values of most parameters still fall behind those for developed countries. China should aim to encourage further improvements, with the findings of these surveys facilitating this campaign.

## Supporting Information

Table S1 **Antimicrobial use prevalence in each department in each survey.**
(DOC)
