# Supplementary material for: Changes in Antimicrobial Use Prevalence in China: Results from Five Point Prevalence Studies
Source: PLoS One. 2013 Dec 23;8(12):e82785. doi: 10.1371/journal.pone.0082785 (PMC3871532; doi:10.1371/journal.pone.0082785)
Supplement: Table S1 — Antimicrobial use prevalence in each department in each survey. (DOC) [file pone.0082785.s001.doc]

Table S1. Antimicrobial use prevalence in each department in each survey

| Departments | Number of surveyed patients (antimicrobial use prevalence; %; 95% confidence intervals) | | | | |
| --- | --- | --- | --- | --- | --- |
|  | 2001 | 2003 | 2005 | 2008 | 2010 |
| **Internal medicine** | | | | | |
| Respiratory medicine | 3589(79.38,78.03-80.67) | 2836(83.00,81.57-84.34) | 4123(75.55,74.21-76.84) | 6863(76.32,75.30-77.31) | 12152(73.23,72.44-74.01) |
| Digestion medicine | 3301(52.56,50.85-54.26 ) | 2891(53.89,52.07-55.70) | 3078(44.12,42.37-45.88 ) | 5975(41.15,39.91-42.40) | 8263(41.14,40.08-42.20) |
| Cardiovascular medicine | 5590(35.92,34.67-37.19) | 3889(36.13,34.63-37.65) | 5178(32.17,30.91-33.46) | 9645(32.40,31.47-33.34) | 15327(29.03,28.32-29.75) |
| Endocrinology | 1689(30.31,28.16-32.54) | 1269(34.52,31.95-37.18) | 2268(25.49,23.74-27.32) | 3356(22.14,20.77-23.58) | 8954(21.58,20.74-22.44 ) |
| Nephropathy medicine | 1034(56.09,53.05-59.09) | 1562(52.82,50.34-55.29) | 1752(43.21,40.91-45.54) | 3176(40.24,38.55-41.96) | 6895( 39.22,38.07-40.38) |
| Infectious diseases | 3156(35.23,33.58-36.91) | 1852(38.17,35.98-40.40) | 3196(36.76,35.11-38.45 ) | 3498(35.16,33.59-36.76) | 8952(43.19,42.17-44.22) |
| Hematology | 2109(48.27,46.14-50.40) | 1598(50.56,48.11-53.01) | 2285(36.11,34.17-38.10) | 2859(35.26,33.53-37.03) | 5475(34.17,32.93-35.44) |
| Neurology | 3566(30.26,28.77-31.79) | 3947(36.48,34.99-37.99) | 5191(20.48,19.40-21.60) | 9589(20.01,19.22-20.82) | 14574(18.98,18.35-19.62) |
| Traditional Chinese medicine | 1981(35.08,33.01-37.21) | 1569(35.18,32.86-37.58) | 1852(28.62,26.61-30.72) | 1978(28.16,26.22-30.18) | 5741( 25.45,24.34-26.59) |
| Others | 3980(41.48,39.96-43.02) | 4658(29.35,28.06-30.67) | 5789(24.89,23.79-26.02) | 8962(24.15,23.28-25.05) | 12747(22.18,21.47-22.91 ) |
| **Surgery** | | | | | |
| General surgery | 6981(70.08,69.00-71.14) | 4759(69.26,67.93-70.55) | 7952(58.69,57.60-59.77) | 13924(56.56,55.73-57.38 ) | 29741( 60.14,59.58-60.70) |
| Thoracic surgery | 2145(68.07,66.07-70.01) | 1658(68.88,66.61-71.06) | 2278(60.49,58.47-62.48 ) | 2889(54.14,52.32-55.95) | 4587(51.45,50.00-52.89) |
| Neurosurgery | 2980(69.50,67.82-71.13) | 2845(59.89,58.08-61.68) | 2989(54.13,52.34-55.91 ) | 5268(49.85,48.50-51.20) | 9563( 47.58,46.58-48.58) |
| Orthopedics | 5892(66,64.78-67.20) | 4658(67.54,66.18-68.87) | 6879(55.24,54.06-56.41) | 13295(49.25,48.40-50.10) | 28071( 50.17,49.59-50.75 ) |
| Surgical urology | 2521(72.23,70.45-73.94) | 1368(72.15,69.72-74.46) | 2989(66.88,65.17-68.54) | 4935(65.53,64.19-66.84) | 9841( 68.47,67.54-69.38) |
| Burns | 687(73.65,70.23-76.81) | 1589(69.86,67.56-72.07) | 895(60.89,57.65-64.03) | 789(59.19,55.72-62.57 ) | 1854( 58.20,55.94-60.43) |
| Plastic surgery | 450(62.44,57.88-66.79) | 289(59.86,54.11-65.35) | 436(50.46,45.78-55.13) | 323(48.30,42.90-53.74 ) | 657( 56.16,52.34-59.91) |
| Oncology | 2100(42.10,40.00-44.22) | 3351(33.24,31.67-34.85) | 3189(26.78,25.27-28.34) | 6989(22.13,21.17-23.12) | 9854( 20.64,19.85-21.45) |
| Others | 1236(58.90,56.13-61.61) | 1578(59.89,57.45-62.28) | 1523(49.57,47.06-52.08) | 3959(46.25,44.70-47.81) | 6574(56.15,54.95-57.35) |
| **Gynaecology** | 2745(69.11,67.36-70.81) | 1978(62.44,60.28-64.55) | 2858(58.68,56.86-60.47) | 5123(56.14,54.78-57.49) | 12574(58.23,57.37-59.09) |
| **Obstetrics** | | | | | |
| Adult group | 1980(66.52,64.41-68.56) | 1657(67.83,65.54-70.04) | 1774(56.99,54.67-59.28) | 3952(58.12,56.57-59.65) | 13487(58.17,57.34-59) |
| Neonatal group | 771(28.15,25.09-31.43) | 852(21.60,18.97-24.49) | 521(17.47,14.45-20.97 ) | 451(15.52,12.47-19.15) | 1245(8.59 ,7.16-10.28 ) |
| **Pediatrics** | | | | | |
| Neonatal group | 501(82.44,78.87-85.52) | 689(83.89,80.96-86.45) | 687(72.63,69.18-75.83) | 1689(78.51,76.49-80.40 ) | 3607( 75.52,74.09-76.90) |
| Non-neonatal group | 2592(81.79,80.26-83.23) | 2123(81.58,79.87-83.17) | 3258(76.58,75.10-78.00) | 5129(80.02,78.90-81.09 ) | 9874( 82.17,81.40-82.91 ) |
| **Ophthalmology and otorhinolaryngology** | | | | | |
| ENT | 2177(70.60,68.65-72.48) | 1968(72.56,70.55-74.49) | 1458(70.23,67.83-72.52 ) | 2241(69.34,67.40-71.21 ) | 6508(67.12,65.97-68.25) |
| Stomatology | 441(73.02,68.69-76.95) | 687(65.94,62.32-69.39) | 589(61.29,57.30-65.14) | 578(59.17,55.12-63.10) | 987( 57.55,54.44-60.60) |
| Ophthalmology | 1890(61.27,59.05-63.44) | 1905(62.47,60.27-64.62) | 1457(48.87,46.31-51.44) | 1962(45.67,43.48-47.88) | 5247( 53.17,51.82-54.52) |
| Others | 92(40.22,30.79-50.44) | 62(45.16,33.42-57.47) | 68(44.12,32.95-55.92) | 251(62.15,56.01-67.92 ) | 528( 76.14,72.32-79.58) |
| **General ICU** | 380(82.89,78.78-86.34) | 350(80.57,76.10-84.37) | 578(82.70,79.40-85.57) | 821(82.70,79.96-85.13) | 1589( 80.18,78.15-82.07) |
| **Other departments** | 2672(38.14,36.32-40.00) | 1689(28.36,26.26-30.56) | 2896(27.87,26.27-29.53) | 3723(21.46,20.17-22.81) | 9874(12.78,12.14-13.45 ) |
